# A99 PHARMACEUTICAL INDUSTRY FUNDING TO GASTROINTESTINAL PATIENT ADVOCACY ORGANIZATIONS

**DOI:** 10.1093/jcag/gwab049.098

**Published:** 2022-02-21

**Authors:** Y Verma, R Bansal, N Gimpaya, M Scaffidi, R Khan, S Grover

**Affiliations:** 1 Division of Gastroenterology, St. Michael’s Hospital, Toronto, ON, Canada; 2 Department of Medicine, University of Toronto, Toronto, ON, Canada

## Abstract

**Background:**

Patient advocacy organizations (PAOs) are not-for-profit organizations that aim to support families and individuals afflicted by illnesses. PAOs play a significant role in guiding health policy, providing education to patients, lobbying, and supporting research. Previous studies have demonstrated that PAOs may receive financial payments from pharmaceutical and medical device manufacturers. This may create a risk of conflict of interest.

**Aims:**

To assess the prevalence and transparency of financial donations from industry to gastrointestinal patient advocacy groups based in the United States (US).

**Methods:**

We conducted a cross-sectional study to determine the prevalence of industry donations to PAOs. Data was extracted from the Kaiser Health News (KHN) Database, a database that tracked payments from pharmaceutical companies to PAOs in 2015. After an initial list of 1215 PAOs was obtained from the database, authors extracted the annual revenues, websites and mission statements for each PAO. Authors individually screened each organization’s mission statement and website to determine whether their primary scope of focus included gastroenterology. A final list of 11 PAOs with annual revenues surpassing $500,000 USD was included for descriptive analysis. From this list, the annual reports and websites of each group were reviewed to determine the extent of transparency of PAOs disclosing financial relationships with industry sponsors. The primary outcome of our study was the total amount of funding that each PAO received from pharmaceutical companies. The secondary outcome was the self-reported amount of funding stated on each PAO’s website and annual report.

**Results:**

From our analysis of 11 PAOs, 9 (81%) organizations received payments from pharmaceutical companies. The median dollar value of donations received was $31,052 USD (IQR=$25 to $302,550). The total dollar value of donations received was $4,059,433 USD. Across the 9 PAOs that received donations, 5 (56%) organizations disclosed a financial relationship with a pharmaceutical company on their website and 2 (22%) disclosed the value of industry donations within a range. No group specified an exact amount of funding received.

**Conclusions:**

Our results demonstrate that a majority of US based gastrointestinal PAOs receive funding from pharmaceutical companies. Furthermore, our results show that many PAOs that receive industry funding do not disclose this amount on their website or annual reports. Given their role in providing patient centered support, it is important for PAOs to disclose financial relationships with industry so as to not produce a conflict of interest.

**Funding Agencies:**

None

